# New rat to mouse xenograft transplantation of endometrium as a model of human endometriosis

**DOI:** 10.1002/ame2.12181

**Published:** 2021-09-03

**Authors:** Amir Abdolmaleki, Cyrus Jalili, Kamran Mansouri, Mitra Bakhtiari

**Affiliations:** ^1^ Department of Anatomical Sciences Faculty of Medicine Kermanshah University of Medical Sciences Kermanshah Iran; ^2^ Medical Biology Research Center Health Technology Institute Kermanshah University of Medical Sciences Kermanshah Iran

**Keywords:** endometriosis, endometrium, mice, rat, xenograft transplantation

## Abstract

**Background:**

Endometriosis can lead to infertility. Since there is no definitive treatment for endometriosis, animal modelling seems necessary to examine the possible treatments. Mouse endometrium cannot be separated for endometriosis induction. In addition, transplantation of uterus into the abdominal viscera to induce endometriosis causes organ damage. In this study, we defined a new model of endometriosis leading to separability of endometrium and a safe anatomical region for transplantation.

**Methods:**

Forty female mice were allocated to 5 groups: 1, sham; 2, allograft uterus transplantation of mice to anterior abdominal wall of mice; 3, allograft uterus transplantation of mice to mesentery of mice; 4, xenograft endometrial transplantation of rat to anterior abdominal wall of mice; 5, xenograft endometrial transplantation of rat to mesentery of mice. Adult female rats with a previous pregnancy experience were selected and placed in the vicinity of male rats for 2 weeks to induce estrogen secretion and increase endometrial thickness.

**Results:**

In the 4th group of animals, compared to sham, the peritoneal concentrations of VEGF‐A, TNF‐α, NO, MDA, and serum levels of CA‐125 and IL‐37 were increased and total body weight was decreased, while weight and size of endometrial lesions were increased significantly (*P* < .05). Genes expression of HOXA10 and HOXA11 were decreased significantly (*P* < .05) in groups 2 and 4 compared to sham.

**Conclusions:**

Xenograft transplantation of endometrium from rat to anterior abdominal wall of mice can potentially mimic human endometriosis morphologically, histologically, and genetically.

## Graphical abstract

In this new model of animal endometriosis, the rat uterus was dissected and grafted to the anterior abdominal wall of mice. The general principles of this method were hormonal and estrous synchronization in transplanted rats and recipient mice, which led to high levels of estrogen secretion in both donor and recipient species. Thus, the thickened endometrial layer in rats was dissected easily and successfully was transplanted and implanted recipient mice. 4 weeks later, all histological, genetic and biochemical factors associated with endometriosis were expressed in transplant recipients.

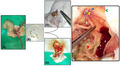

## 1 INTRODUCTION

Endometrium is the inner epithelial layer of mammalian uterus and is divided into thin basal and thick functional layers. The presence of a functional layer strictly depends on the menstrual (in humans) or estrous (in rodents) cycles.

Endometriosis is a pathological condition in which the endometrial cells grow outside the uterus in abdominal or pelvic cavities. These cells reside on the peritoneum layer located on organs (such as ovaries, fallopian tubes, uterus, and intestine), attract adjacent blood vessels (angiogenesis), and grow by triggering inflammation. These pathological conditions lead to formation of new endometrial lesions. Thus, angiogenesis and inflammation are two crucial biological phenomena in endometriosis. Complications of this disease include formation of scar tissue, organ adhesions, pus‐filled cysts, and adhesion‐related obstruction (in intestines or uterus lumina). In addition, following scar formation, anatomical disposition, and uterus obstruction, the occurrence of female infertility is probable, and is considered an end‐stage outcome of endometriosis.^1^

The most common theory of human endometriosis is that endometrial cells follow a retrograde route from the intrauterine cavity to the peritoneal fossa. Although most studies in the field of infertility and reproduction are performed in mouse models rather than other animals, the presence of intrinsic endometriosis in mice is impossible because there are no menstrual cycles and endometrium abscission in rodents and these integrated cells are absorbed by adjacent cells. Thus, in mice induction of endometriosis via a surgical procedure is necessary to assess aspects of human endometriosis.^2^

The most common animal models for endometriosis are rodents, monkeys, and rabbits.^3^ In 1985 Vernon introduced a rat model of endometriosis through transplantation of uterus fragments to the peritoneal layer,^4^ and in 1995 Cummings achieved a mouse model of endometriosis through transplantation of uterus to the mesentery layer.^5^ In these models uterus fragments were used for endometriosis induction, but based on the histopathology of endometriosis, this pathological condition is caused by the attachment of endometrial cells to the peritoneal layer. Thus, it seems that isolation of the endometrium from the two other layers of the uterus (myometrium and perimetrium) can probably model human endometriosis conditions more closely.^6^ Endometrium in mice is a thin microscopic layer that is often impossible to dissect out, and besides the endometrium is a fragile tissue which cannot be sutured. However, the endometrial layer in the proliferative phase of of the estrous cycle is thick enough to be dissected and transplanted. In addition, transplantation of endometrium to vital organs such as mesentery, as a routine protocol of endometriosis induction, can potentially damage vessels, leading to internal hemorrhage and animal death. These difficulties make endometriosis induction a complicated process in mice.

Thus developing a new animal endometriosis model and finding a suitable anatomical site for endometrial implantation that completely mimics human endometriosis conditions, with fewer complications, seems necessary. In the present study, we compared three models of animal endometriosis (rat‐rat and mouse‐mouse allografts, and rat‐mouse xenografts) with transplants to the anterior abdominal wall and mesentery layer. In this experimental animal study, we assessed the recipient animals using various biomarkers, including rate of angiogenesis (serum levels of VEGF‐A and vessels count), serum levels of CA‐125, inflammatory biomarkers (TNF‐α in peritoneal fluid and IL‐37 in blood serum), oxidative stress biomarkers (NO and MDA in peritoneal fluid) and histological assessments to ensure that the new model accurately imitated human endometriosis.

## 2 METHODS

### 2.1 Laboratory animals and ethical approval statements

Forty female adult mice (NMRI, 25‐30 g, 8‐12 weeks) were purchased from the animal house of the university. To acclimatize to the new living environment, the animals were kept in the animal house for 2 weeks with no experimental treatments. Standard living conditions (22 ± 2°C, 12‐hour light/12‐hour dark, and free access to water and food pellets) were provided for all animals. All procedures performed in the study were in accordance with the ethical standards of the institutional and/or national research committee and with the 1964 Helsinki Declaration. This study complied with ethical and humane principles of research and was approved by the Ethics Committee of the Kermanshah University of Medical Sciences (Ethics Code: IR.KUMS.REC.1399.994).

### 2.2 Study animal groups

Forty female mice were divided into 5 experimental groups (n = 8 in each group): 1, sham (application of laparotomy followed by abdominal wall suture with no experimental treatments); 2, mouse‐mouse allograft – mouse uterus transplantation to mouse anterior abdominal wall; 3, mouse‐mouse allograft – mouse uterus transplantation to mouse mesentery; 4, rat‐mouse xenograft – rat endometrial transplantation to mouse anterior abdominal wall; 5, rat‐mouse xenograft – rat endometrial transplantation to mouse mesentery. In all treatment groups, the laparotomy was applied in the ventral midline region of the animals. Due to the thinness and inseparability of mouse endometrium, all layers of mouse uterus (endometrium, myometrium, and perimetrium) were cut and transplanted as allografts. While in rats, the endometrium was separated from total uterus tissue and transplanted to recipient mice in a xenograft procedure. Two anatomical transplantation locations were used: anterior abdominal wall and mesentery of small intestine. In all sites of transplantation, the uterine epithelium was sutured directly to the peritoneal layer.

### 2.3 Preparation of donor animals

To increase the endometrium thickness in donor rats and facilitate endometrial dissection, a principle modification in this method, three main conditions were established: all female rats or mice were of adult age (to increase their uterine response sensitivity to proliferation following estrogen secretion), all animals had a previous pregnancy experience (to produce separatable endometrium from other uterine layers in the xenograft procedure), and the animals were exposed to male animals for 2 weeks (a ratio of one male/two females for sexual stimulation). Animals were housed in special plastic cages divided by a metal grid to prevent mating. This indirect contact caused visual sexual stimulation, leading to estrogen secretion in donor female rats or mice. Also, the animals’ straw was not replaced with a new bedding while they were in the cages, which resulted in pheromone secretion and urine odor. These special conditions caused sexual stimulation in female rats or mice leading to estrogen secretion and endometrial thickening.^7^

### 2.4 Hormonal and estrous cycle synchronization in recipient animals

According to the Whitten effect and based on the estrogen‐dependent nature of endometriosis in rodents, the female recipient mice were exposed to males (indirectly through metal nested cages). The female mice were housed in the vicinity of males in special cages divided by metal grids before (2 weeks) and during (4 weeks) endometrial induction. As with donor animals, these conditions caused the estrogen secretion necessary for successful implantation of endometrial fragments. Also, for estrus cycle synchronization, vaginal cytology was checked daily for a week before the surgery. A cotton swab impregnated with normal saline was used to collect a vaginal smear from the vaginal orifice. The samples were stained using the Papa‐Nicola staining method, and endometriosis induction was performed during the estrus stage for all animals. The mice were sexually receptive in estrus cycle as characterized by estrogen secretion. In this study, the estrus cycle was detected by the presence of whole superficial cells of different types during microscopic examination of vaginal cytology.^8^

### 2.5 Implantation of endometrium to abdominal wall and mesentery

**FIGURE 1:**
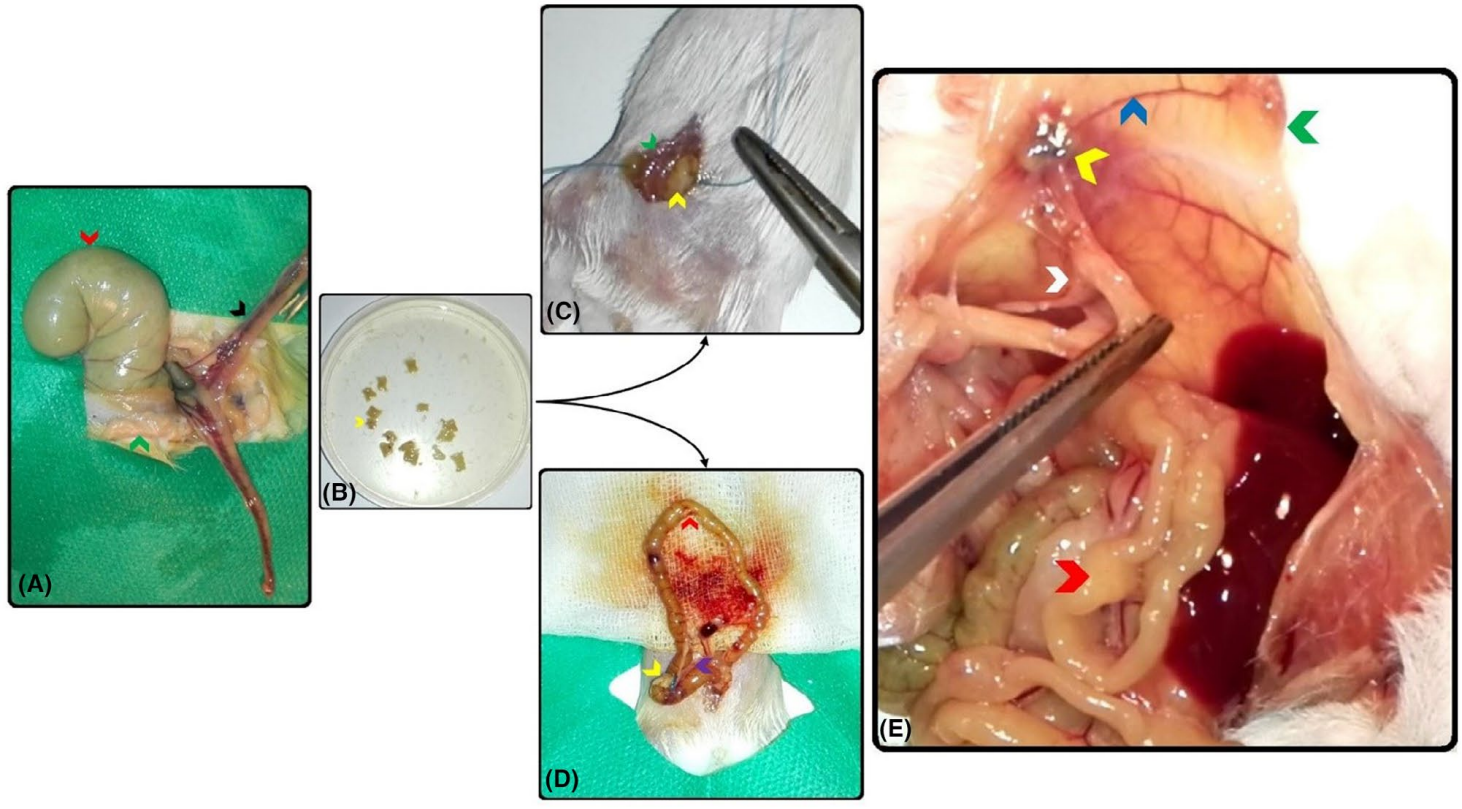
Rat‐mouse xenograft transplantation of endometrial segments (A and B) to abdomonal wall (C) and small intestine mesentery (D) for endometriosis induction (E). Arrows represent intestine (red), uterus horn (black), abdominal wall (green), angiogenesis (blue), mesentery artey (purple), endometrial lesions (yellow), and endometrial tissue attachments (white)

Donor animals (mice and rats) were anesthetized through intraperitoneal injection of 25 IU ketamine‐xylazine/25g animal (10 IU ketamine/90 IU xylazine) and then they were euthanized through cervical dislocation. Uterus (of mouse) and endometrium (of rats) were dissected, and all surrounding attached connective tissues were removed under a loop microscope (Figure 1A). Dissected tissues were placed in DMEMF12/FBS 5% cell culture solution to preserve cell viability (Figure 1B). Round grafts were prepared using a 3‐mm diameter punch and were sutured (nylon, 5‐0 USP, SUPA medical devices, Iran) to the anterior abdominal wall or mesentery layer of small intestine in recipient animals (Figure 1C,D). Recipient mice were weighed and anaesthetized with 25 IU ketamine‐xylazine/25 g animal. The small intestine was explored, and the inner epithelial surface (containing endometrium) of grafts was sutured in direct contact with the peritoneal surface of mesentery or abdominal wall. Also, for preparation of blood supply, the grafts were transplanted as close as possible to mesentery vessels. Peritoneal and muscular layers of abdominal wall were sutured using absorbable threads (chromic 5‐0 USP, SUPA medical devices, Iran), and the skin was closed with nylon (Nylon, 5‐0 USP, SUPA medical devices, Iran). A day after recovery from the surgery, the recipient animals were exposed to males (for 4 weeks) in separate metal cages (to prevent mating) for endometriosis induction (Figure 1). Prior to tissue implantation, the size and weight of whole grafts were recorded.^9^

### 2.6 Animal weighing, dissection, and tissue sampling

Four weeks after surgery, the recipient mice were anesthetized and then were euthanized by cervical dislocation procedure. Immediately, 1 mL of injectable distilled water was injected into the peritoneal cavity. Two minutes later, the peritoneal fluid was aspirated. After laparotomy, the endometrial lesions and uterus were dissected. Thoracotomy was also performed, and blood was aspirated from the right ventricle and centrifuged (3000 *g*, 15 min) to separated blood serum. All biological samples were frozen in liquid nitrogen for future biochemical and genetic analysis or were fixed in 10% formaldehyde for histopathological assessments. Total body weight was also recorded.

### 2.7 Morphometric assessments of endometrial lesions

During the tissue sampling procedure, the diameter of endometrial lesions was calibrated (Calliper, Sana't Co., Iran), and the weight of the grafts was calculated (Laboratory scale, Model GR 202, Tajhizat Co., Iran) after complete excision of surrounding connective tissues. The presence or absence of pus‐filled cysts was also recorded. These factors were considered as growth markers of endometrial lesions, and they were compared with the primary size (3 mm) and primary weight (0.01 g) of endometrial lesions exactly prior to implantation.

### 2.8 Assessment of angiogenesis in endometrial lesions and peritoneal fluid

To evaluate angiogenesis rate in endometrial lesions, the number of newly generated vessels around endometrial lesions were counted using histopathological sections (Nicon biology microscope, E200). Also, the peritoneal concentration of VEGF‐A, a peritoneal angiogenic biomarker, was assessed biochemically using an ELISA kit (Abcam, ab100662, USA) according to routine procedure based on the manufacturer's instruction.^10^

### 2.9 Assessment of inflammation status in peritoneal fluid and blood serum

The concentration of TNF‐α (Abcam, ab193687, USA) in peritoneal fluid was considered a marker of inflammation status induced by endometrial lesions and produced by peritoneal macrophages. IL‐37 (Abcam, ab213798, USA) was also measured as a serum biomarker for endometriosis diagnosis. These measurements were done using an ELISA kit according to the manufacture's protocol.^10^

### 2.10 Status of oxidative stress in peritoneal fluid following endometriosis induction

Generated oxidative stress following hyper‐activation of macrophages and high rate proliferation of endometrial cells was measured in peritoneal fluid using an ELISA kit. In this process, NO concentration (Abcam, ab272517, USA) was evaluated using the Griess test, and MDA (Abcam, ab238537, USA) levels (representing lipid peroxidation status) were also measured.^10^

### 2.11 Serum levels of CA‐125

Cancer antigen 125 is a member of the mucin family of glycoproteins. CA‐125 is a biomarker which is elevated in the blood of some types of cancers and endometriosis. This factor was measured using an ELISA kit (Abcam, ab108653, USA).^10^

### 2.12 Histopathological assessments using H&E and Perls staining

The right horn of uterus and half of endometriosis lesions were fixed in 10% formaldehyde for H&E and Perls staining. H&E staining was used for glandular and stromal assessments of endometriosis, and Perls staining was also used to assess hemosiderin deposition in macrophages as an accepted factor for confirming endometriosis. Tissue processing was performed, and paraffin blocks were prepared. Thin sections (5 µm) were cut (Microtome, Leica RM 2125, Germany) and stained using H&E and Perls staining. Finally, the slides were assessed using a research microscope (Olympus, BX‐51T‐32E01) based on histopathological variations in the tissue including epithelium and stroma of endometrium, endometrial glands, blood vessels, and macrophages loaded with hemosiderin.^10^

### 2.13 RNA extraction and real‐time quantitative PCR of HOX genes expression in uterus

Variation of HOX genes (HOXA 10 and 11) in endometriosis is an established indicator of embryo implantation during fertility according to the literature. In order to assess the probable pathological effects of endometriosis on uterus, the gene expression of HOXA10 (F: GCCCTTCCGAGAGCAGCAAAG, R: AGGTGGACGCTGCGGCTAATCTCTA) and HOXA11 (F: GATTTCTCCAGCCTCCCTTC, R: AGAAATTGGACGAGACTGCG) were assessed. Left horn of uterus was dissected, and total RNA was extracted (QIAGEN RNA purification kit). The quality of the extracted RNA was checked by measuring the 260/280 nm wavelength absorbance ratio with a spectrophotometer (UV1240, Shimadzu, Kyoto, Japan). DNA was synthesized using a commercial BioFact kit (BioFact RT Series, Korea). Gene expression was evaluated using High ROX BioFact™ 2X Real‐Time PCR Smart mix SYBR Green PCR master mix, with β‐Actin (F: GGCACCACACCTTCTACAATG, R: GGGGTGTTGAAGGTCTCAAAC) used as a housekeeping gene. Gene expression levels were measured using the Ct (2^−ΔΔt^) method (fold changes).

### 2.14 Statistical analysis

After data extraction, the Kolmogorov–Smirnov test was first conducted to confirm data compliance with normal distribution. One‐way analysis of variance (one‐way ANOVA) was used for statistical analysis, and the Tukey post hoc test was used to determine the difference between the groups. Statistical Package for the Social Sciences 16 (SPSS Inc, Chicago, IL) was used for data analysis, the results were expressed as mean ± SD, and *P* < .05 was considered significant.

## 3 RESULTS

### 3.1 Angiogenesis rate

The angiogenesis rate was assessed by measuring the peritoneal concentration of VEGF‐A and vessel counts in microscopic sections. The peritoneal fluid concentration of VEGF‐A was raised significantly (*P* < .05) in mouse‐mouse allograft and rat‐mouse xenograft transplantations compared to the sham group. The levels of VEGF‐A were also significantly raised (*P* < .05) in rat‐mouse xenograft groups compared to mouse‐mouse allograft animals. In addition, newly formed vessels were significantly (*P* < .05) increased in all groups of allograft and xenograft transplantations compared to the sham group. No significant (*P* > .05) differences were detected in vessel count in xenograft animals compared to allografts (Table 1).

**TABLE 1:** Rate of angiogenesis, inflammation, CA‐125, oxidative stress, and alteration of physical features in sham and various transplanted groups

Group no	Angiogenesis		CA‐125 (pg/mL)	Inflammation		Oxidative stress		Physical features		
	VEGF‐A (pg/mL)	Vessel count		TNF‐α (pg/mL)	IL‐37 (pg/mL)	NO (OD%)	MDA (nm/mg)	Total body weight (g)	En lesions weight (g)	En diameter (mm)
1	190.1 ± 7.02	0	8 ± 0.2	1.1 ± 0.2	37 ± 4.1	4.8 ± 0.7	2.1 ± 0.3	25.8 ± 2.4	0	0
2	432 ± 43.5*	8 ± 2*	34.01 ± 1.2*	3.8 ± 0.9*	54.02 ± 3.4*	7.3 ± 1.7*	8.3 ± 1.1*	21.2 ± 2.2*	0.8 ± 0.01^ɷ^	8 ± 0.1^ȸ^
3	398 ± 21.1*	5 ± 1*	29.4 ± 2.3*	3.3 ± 1.01*	48.2 ± 2.9*	6.2 ± 0.9*	6.2 ± 2.01*	24 ± 1.3	0.19 ± 0.1^ɷ^	5 ± 0.2^ȸ^
4	512 ± 25.8* ^,^ ^ɸ^	9 ± 2*	35.8 ± 1.6*	3.5 ± 1.2*	68.3 ± 4.1* ^,^ ^ɸ^	7.4 ± 2.2*	8.9 ± 2.4*	19.8 ± 2.3*	1.8 ± 0.04^ɸ^, ^ɷ^	12 ± 0.03^ɸ^, ^ȸ^
5	490 ± 50.4* ^,^ ^ɸ^	4 ± 1*	28.2 ± 0.9*	2.8 ± 0.4*	59.1 ± 3.3* ^,^ ^ɸ^	6.8 ± 109*	5.9 ± 1.4*	23.6 ± 2.6	0.70 ± 0.01^ɷ^	4 ± 0.1^ȸ^

Note

Exprimental groups; 1, sham; 2, mouse‐mouse allograft – uterus transplantation to anterior abdominal wall; 3, mouse‐mouse allograft – uterus transplantation to mesentery; 4, rat‐mouse xenograft – endometrial transplantation of rat to anterior abdominal wall of mouse; 5, rat‐mouse xenograft – endometrial transplantation of rat to mesentery of mouse. Data are presented as mean ± SD.

Abbreviations: CA‐125, cancer antigen 125; En, endometriosis; IL‐37, interleukin 37; MDA, malondialdehyde; NO, nitric oxide; TNF‐α, tumor necrosis factor α; VEGF‐A, vascular endothelial growth factor A.

* Indicates *P* < .05 compared to sham group.

^ɸ^ Indicates *P* < .05 compared to allograft mouse‐mouse groups.

^ȸ^ Indicates *P* < .05 compared to the primary size of endometrial lesions (3 mm) prior to implantation.

^ɷ^ Indicates *P* < .05 compared to the primary weight of endometriosis lesions (0.01 g) prior to implantation.

### 3.2 Serum concentration of CA‐125

CA‐125 biomarker, which represented the cancer antigen in blood serum, was significantly (*P* < .05) elevated in all allograft and xenograft transplantations in comparison with sham animals, while no significant (*P* > .05) difference was found between the rat‐mouse xenograft transplantation groups and the allograft groups (Table 1).

### 3.3 Inflammation status

Inflammation was assessed using the peritoneal concentration of TNF‐α and serum levels of IL‐37 as crucial inflammatory markers in endometriosis. In all treatment groups (allografts and xenografts), serum levels of TNF‐α in peritoneal fluid were increased significantly (*P* < .05) compared to sham group, and non‐significant (*P* > .05) differences in TNF‐α concentration were detected between xenograft animals and allograft mice. Serum levels of IL‐37 were increased significantly (*P* < .05) in all animals in allograft and xenograft transplantation groups in comparison with sham animals. Significant (*P* < .05) incremental changes in IL‐37 levels were also seen between rat‐mouse xenograft groups and mouse‐mouse allograft animals (Table 1).

### 3.4 Oxidative stress status

The concentration of all oxidative stress markers (NO and MDA) was increased significantly (*P* < .05) in whole transplanted animals (both allografts and xenograft treatment groups) compared to the sham group. No significant (*P* > .05) alteration was found among the animals in xenograft groups compared to the allograft groups (Table 1).

### 3.5 Physical features

Following endometriosis induction, total body weight of recipient animals decreased significantly (*P* < .05) in the 2nd (mouse‐mouse allograft – uterus transplantation to anterior abdominal wall) and 4th (rat‐mouse xenograft – endometrial transplantation of rat to anterior abdominal wall of mouse) groups of treatment in comparison with the sham group. In other transplanted groups (to serous mesentery layer), non‐significant (*P* > .05) changes in the animals’ weights were observed compared with the sham group. The weight of endometrial lesions was also increased significantly (*P* < .05) in allograft and xenograft mice at the end of surgery compared to the primary weight (0.01 g) before endometriosis induction. The weight endometrial lesions in animals in the 4th treatment group (rat‐mouse xenograft – endometrial transplantation of rat to anterior abdominal wall of mouse) showed a significant (*P* < .05) increase compared to animals with allograft transplantations. Significant (*P* < .05) increases in the diameter of lesions was found in all animals with allograft and xenograft transplanted tissues compared to the primary size of lesions prior to transplantation (3 mm). More obviously, the size of the transplanted endometrial lesion increased significantly (*P* < .05) in rat‐mouse xenograft transplantation to anterior abdominal wall compared to allografts (Table 1).

### 3.6 Histopathological variations

**FIGURE 2:**
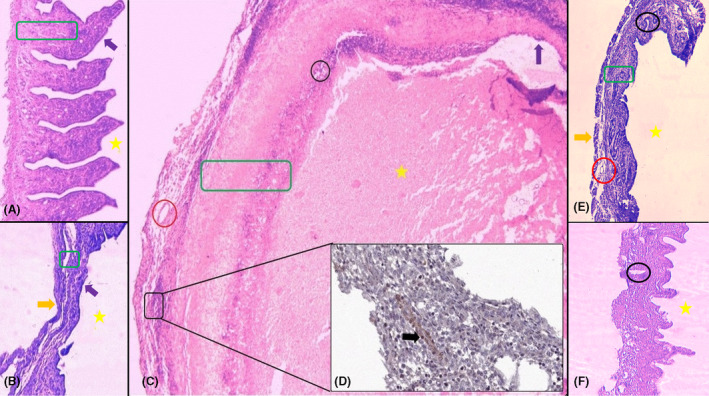
Histological H&E sections of endometriosis in various groups. Thickened endometrium prior to implantation (A), allograft mouse‐mouse uterus transplantation to anterior abdominal wall (B), xenograft rat‐mouse endometrium transplantation to anterior abdominal wall (C), Perls staining of hemosiderin‐containing macrphages (D), allograft mouse‐mouse uterus transplantation to mesentery layer (E), xenograft rat‐mouse endometrium transplantation to mesentery layer (F). Epithelium (purple arrows), stroma (green rectangles), sections of endometrial glands (black circles), lumen of endometrial cyst (yellow stars), blood vessels (red circles), myometrium and perimetrium layers (orange arrows), and hemosiderin deposit in macrophages (black arrow). H&E (100×) and Perls staining (400×)

As depicted in Figure 2, the endometrial grafts of rats (with dissected layers of myometrium and perimetrium) were stained using H&E prior to implantation. Obvious epithelium (purple arrow) with compact stromal tissue (green rectangular) was seen located above lamina properia layer (Figure 2A). Also, stroma had coronal sections of endometrial glands. After implantation of mouse uterus to abdominal wall of recipient mice (Figure 2B), pathological features were found including: epithelial layer (Figure 2B, purple arrow) with no villi, stroma (Figure 2B, green rectangular) seen as a thin layer with multiple cavities, and fewer of coronal sections of blood vessels. Also, as shown in Figure 2B, the thick layer of myometrium and perimetrium degenerated following uterus transplantation (orange arrow). These histopathological changes led to less secretion of pus in the luminal space of endometrium, decreased size of grafts, and reduced weight of lesions after a 4‐week implantation. Figure 2C shows a complete section of rat‐mouse endometrial xenograft transplantation to abdominal wall. Although the endometrial layer showed an irregular pattern compared to normal histology, thick and glandular epithelial formation caused the generation of a lumen full of secretions and hemorrhages (Figure 2C, yellow star). The outer part of this type of endometrial transplantation (xenograft rat‐mouse implantation) had a thick attachment layer as a common feature of endometriosis (Figure 2C) with many coronal sections of blood vessels (Figure 2C, red circle). There were many glandular sections (black circle) within the thickness of the stromal layer (Figure 2C, green rectangle). Figure 2D presents Perls staining of a section of connective tissue around endometrial lesions containing blood vessels. The bBlack arrow (Figure 2D) shows macrophages loaded with hemosiderin following hemorrhages in endometrial lesions of xenograft transplantation to anterior abdominal wall. In mouse‐mouse transplantation of uterus to mesentry (Figure 2E), myometrium and perimetrium layers were found (orange arrow) containing sections of blood vessels in perimetrium. Routine and regular stromal glands (black circle) were detected but with less pus secretion in the luminal cavity (yellow star). Figure 2F represents xenograft transplantation of endometrium to mesentery layer. In these histological sections, many glandular lumina (black circle) were found with no pus in the cavity (yellow star).

### 3.7 HOX gene expression in uterus of recipient animals

**FIGURE 3:**
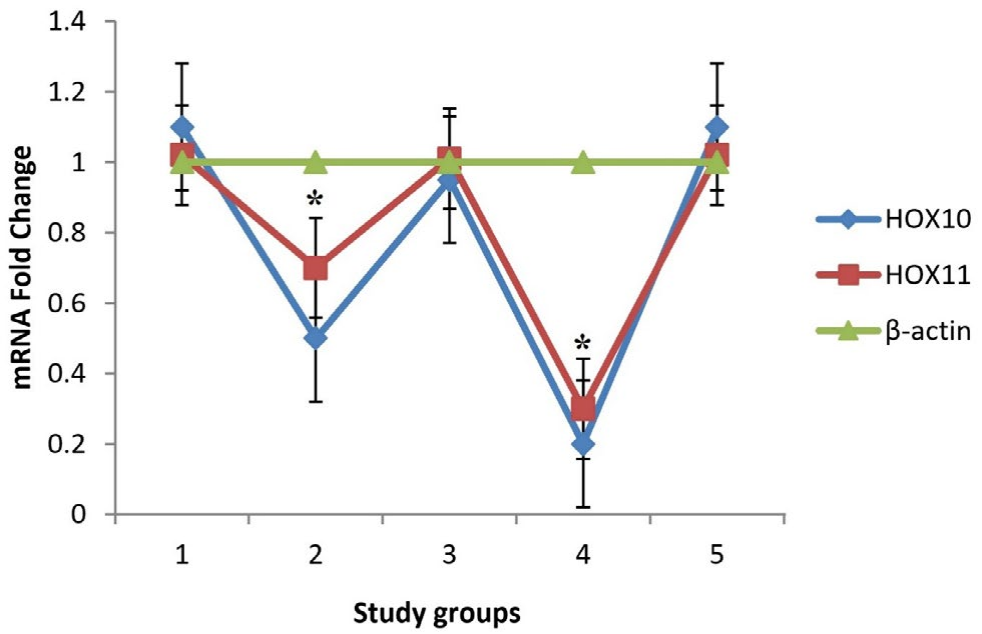
Rate of genes expression in uterus of graft‐recipient animals. Exprimental groups: 1, sham; 2, mouse‐mouse allograft – uterus transplantation to anterior abdominal wall; 3, mouse‐mouse allograft – uterus transplantation to mesentery; 4, rat‐mouse xenograft – endometrial transplantation of rat to anterior abdominal wall of mouse; 5, rat‐mouse xenograft – endometrial transplantation of rat to mesentery of mouse. Data are presented as mean ± SD. *P < .05 compared to β‐actin baseline

Following induction of endometriosis in recipient groups 2 (mouse‐mouse allograft uterus transplantation to anterior abdominal wall) and 4 (rat‐mouse xenograft endometrial transplantation of rat to anterior abdominal wall of mouse), significantly (*P* <.05) decreased levels of HOXA10 and HOXA11 genes expression were found. In other treatment groups, no significant (*P* > .05) alterations in genes expression of HOXA10 and HOXA11 were detected compared to the β‐actin baseline (Figure 3).

## 4 DISCUSSION

In this experimental study, we present a xenograft (rat to mouse) model of endometriosis through implantation of endometrium of rat to anterior abdominal wall of mouse. In the study, estrogen production was stimulated, leading to increased endometrium thickness, through exposure of adult female rats (with previous pregnancy experience) to males (for 2 weeks). The recipient mice were also stimulated (using exposure to males) to produce estrogen to enable successful transplantation of endometrial grafts. Finally, we found that due to the estrous cycle and induction of estrogen production in donor rats, it is possible to increase the thickness of the rat endometrium, which can then be dissected easily. Implantation of these dissected lesions to the anterior abdominal wall of recipient mice can lead to successful endometrial induction with no visceral damage.

Two of the main features of endometriosis are angiogenesis (to preserve the endometrial fragments viability) and inflammation (for successful implantation of endometrial lesions). Macrophages located in the peritoneal layer are essential elements involved in the onset of inflammation leading to release of several inflammatory factors.^11^ TNF‐α is an important initiator of inflammation and is secreted by peritoneal macrophages into endometrial lesions and peritoneal fluid. Thus, allograft or xenograft endometrial transplantations can provide acceptable inflammation to initiate a successful endometriosis induction. In a meta‐analysis study, Cao and coworkers investigated the association of TNF‐α gene T‐1031C polymorphism with endometriosis. Much as we found high levels of TNF‐α in our study, they also concluded that TNF‐α is an important factor in advancement of endometriosis.^12^ Although their study considered only human cases; the final result was parallel to our findings of an increase in peritoneal levels of TNF‐α in animals with endometriosis. TNF‐α can also induce the proliferation, differentiation, and migration of endometrial cells during endometriosis. Thus, it has been proposed that blocking TNF‐α activity can potentially reduce development of endometriosis.^13^ IL‐37 is another essential blood factor that indicates the presence of inflammation such as endometriosis, as we found in this study. The levels of IL‐37 in blood serum correlate with endometriosis severity.^14^ In our experimental study, we found that high levels of IL‐37 are more prominent in xenograft transplanted animals compared with other allografts. Jiang and coworkers introduced IL‐37 as a biomarker for endometriosis diagnosis because they found the increased levels of this biomarker in human endometriotic cases.^15^

Angiogenesis is another critical factor in successful induction of endometriosis. In this study, the rate of angiogenesis was determined from two markers, VEGF‐A and the number of blood vessels per unit area of histological sections. VEGF‐A, a glycosylated mitogen, has proliferative effects on endothelial cells leading to angiogenesis. The presence of macrophages at the site of vascular lesion transplantation has been suggested as a source of VEGF‐A secretion. Thus, through initiation of inflammation following transplantation of endometrium, angiogenesis subsequently occurs following VEGF‐A secretion by macrophages. The findings of biochemical and microscopic studies show that in xenograft transplant models a high rate of angiogenesis and factors involved in this process are observed. As Takehara and coworkers found, high levels of VEGF‐A were also detected in peritoneal fluid. The results of Takehara`s study suggested that endometriosis may arise from eutopic endometrium with higher levels of angiogenic activity possibly induced by VEGF‐A.^16^

CA‐125 is one of the surface components of epithelia in female reproductive tract. This protein is highly glycosylated, creating a hydrophilic barrier.^17^ CA‐125 has been shown to play a role in advanced tumorigenesis and tumor proliferation. This factor is considered a serum biomarker of endometriosis.^18^ The expression of CA‐125 is altered in several types of cancers and endometriosis.^19^ Since this protein is located on the cell surface of endometrial lesions, thus in late stage of endometriosis, serum levels of this protein were elevated.^20^ In the present study, significantly increased serum levels of CA‐125 was also found in all cases of endometriosis (including allografts and xenografts). CA‐125 also acts in cell‐to‐cell interactions for metastasis of cancerous tumors or proliferation of transplanted masses. This factor selectively binds to mesothelin (a glycoprotein expressed by mesothelial cells of peritoneum) to be implanted into the peritoneal layer.^21^ Rokhgireh et al evaluated the potential use of CA‐125 assessment as a non‐invasive protocol for endometriosis diagnosis. They concluded that following endometriosis induction, high levels of CA‐125 in blood serum were also likely to be found, which is parallel to our findings.^22^

Recent studies have focused on the role of oxidative stress on endometriosis pathophysiology causing a general inflammatory response in the endometrial implanted region. Reactive oxygen species (ROS) are physiological inflammatory mediators modulating cell proliferation; besides, they have deleterious effects in uncontrolled activity or high concentrations. The connection between endometrial cell proliferation and ROS production is widely accepted and has been well studied.^23^ Peritoneal aggregation of macrophages occurs at a higher rate in women with endometriosis. Macrophages release prostaglandins, cytokines, and growth factors leading to a high production of ROS. Osborn et al assessed the expression of NO synthase by peritoneal macrophages in endometriosis‐associated infertility. Osborn concluded that in addition to the effects of NO on reproduction, peritoneal NO levels could potentially induce inflammation in women with endometriosis.^24^ MDA, a marker for oxidative stress, results from lipid peroxidation of polyunsaturated fatty acids. ROS degrades polyunsaturated lipids, forming MDA. This compound is a reactive aldehyde and is one of the many reactive electrophile species causing detrimental cellular stress. Thus, the level of MDA production is considered a biomarker for measuring the level of oxidative stress in the peritoneal cavity following endometriosis induction.

According to published articles, physical changes following endometriosis induction are likely in humans, such as decreased total body weight, increased size of endometriosis lesions, and weight acceleration of endometrial lesions. Amaral et al developed an experimental model of endometriosis in rats. They implanted total sections of uterus in abdominal wall. Contrary to our findings, they found non‐significant differences in the total weight of the animals.^25^ It seems that attachment of endometrial lesions to the anterior wall of the abdomen initiates all the processes of inflammation and angiogenesis, leading to the maximal growth of lesions. Finally, these molecular mechanisms cause animal weight loss via an unknown mechanism. In addition, microscopic and macroscopic examinations of endometriosis lesions have shown that angiogenesis occurring in lesions attached to the anterior abdominal had more blood supply than the lesions attached to the mesentery. This finding probably explains the extended growth of lesions attached to the anterior abdominal wall where a better blood supply results in increased size and weight of lesions compared to other animals with the endometrium attached to the mesentery.

Following transplantation of xenograft endometrial tissue to the anterior abdominal wall of recipient mice, the normal physiological arrangement of epithelium was disrupted, and a pathological form developed that included irregular epithelium formation and a non‐columnar epithelial layer with high pus secretion. The high proliferation rate of stromal cells also covered most of the thickness of grafts with different irregular sections of endometrial glands. In allograft endometriosis lesions, a thin layer of primitive and immature epithelium and a thin and degenerative stroma were detected. The shrunken stroma following uterine allograft transplantation contained numerous gaps instead of typical endometrial glands. Examination of the outer layer of grafted specimens showed that transplantation of xenograft tissues to the anterior wall of the abdomen could induce proliferation of connective tissue in the form of scars or extensive dissectible adhesions, while in the allograft groups, although the uterus was transplanted with all layers (endometrium, myometrium, and perimetrium), the myometrium and perimetrium layers degenerated. Besides, although the myometrium and perimetrium layers were dissected out in xenograft transplantation, extensive outer connective tissue was generated. The presence of blood vessels in the connective tissue led to a vast hemorrhage in endometriosis lesions which was detected by Perl staining. During this stage, hemosiderin granules were deposited in macrophages, detected by pearl staining against Fe atoms.

In late stages of endometriosis, the gene expression of HOX in uterus can be altered. HOXA10 and HOXA11 are homeobox genes that act as transcription factors essential to embryonic development. The role of each of these two genes in regulation of endometrial development has been proved.^26^ Aberrant HOX gene expression following endometriosis contribute to the aetiology of female infertility in these patients. In the present study, gene expression of HOXA10 and HOXA11 in uterus tissue were decreased in all allograft and xenograft transplanted to the anterior abdominal wall. Altered gene expression in uterus is considered as the main factor in the end stage of endometriosis pathology.

This study aimed to determine the animal model most similar to human endometriosis with regard to molecular, histological, and genetic characteristics. The main modifications to the protocol for using rats as graft donor to accelerate the success rate of xenigraft implantation were: previous pregnancy experience, and exposure to male animals for 2 weeks. The graft recipient mice were also exposed to males for 4 weeks. These essential conditions led to increased estrogen levels and greater thickness of endometrium for effective dissection and successful endometrial implantation. Endometrial lesions isolated from stimulated uterus of a rat implanted in the anterior abdominal wall of mice in a xenograft surgical process could potentially be an appropriate option for endometriosis modelling in rodents. For future studies, additional laboratory assays such as immunoassay and protein immunoblotting are strongly recommended. Also, inclusion of a human endometriosis sample to compare with rodent endometriosis lesions will be necessary to upgrade the level of study.

## CONFLICT OF INTERESTS

The authors declare that they have no competing interests.

## AUTHORS’ CONTRIBUTIONS

M.B A.A conceived and designed the study, supervised the data collection, interpreted the results, and revised the manuscript. A.A conducted the experimental procedures, data collection analysis, and manuscript preparation. C.J was a scientific advisor for conducting laboratory analysis. K.M was a scientific advisor in the filed of angiogenesis pathways and animal disease modelling. All authors read and approved the final manuscript prior to submission.

## ETHICS APPROVAL AND CONSENT TO PARTICIPATE

All assessments were conducted on in accordance with ethical principles and under the supervision of the University's Ethics Committee (Ethic NO. IR.KUMS.REC.1399.994). The study was reviewed by the appropriate ethics committee and had been performed in accordance with the ethical standards described in an appropriate version of the 1975 Declaration of Helsinki, as revised in 2000.

## CONSENT FOR PUBLICATION

Not applicable.

## Data Availability

The datasets used and analyzed for this are available from the corresponding author upon reasonable request.
